# Psychostimulant Prescription in Children and Adolescents With Attention Deficit Hyperactivity Disorder: Evaluating the Current Practice in Al Ain, United Arab Emirates

**DOI:** 10.1192/bjo.2024.481

**Published:** 2024-08-01

**Authors:** Syed Fahad Javaid, Aysha Ali Al Ahbabi, Ayesha Afzal, Noura Ali Al Ahbabi, Mohammed Ali Al Ahbabi

**Affiliations:** 1 Department of Psychiatry and Behavioral Sciences, College of Medicine and Health Sciences, United Arab Emirates University, Al Ain, UAE; 2 Tawam Hospital, Al Ain, UAE; 3 Behavioral Sciences Institute, Al Ain Hospital, Al Ain, UAE

## Abstract

**Aims:**

Attention Deficit Hyperactivity Disorder (ADHD) is a neurodevelopmental disorder characterised by the core symptoms of hyperactivity, impulsivity, and associated functional impairment. Psychostimulants are the most commonly prescribed medication for ADHD in children and adolescents. The use of psychostimulants in children and adolescents requires close supervision in a specialist clinic. The decision to commence psychostimulants should be made jointly with the young person, their parents, carers, and healthcare professionals. It is critical to provide age-appropriate information and discuss treatment's possible benefits and side effects.

This audit aimed to appraise the psychostimulant prescribing practice in children and adolescents in psychiatric outpatient clinics in Al Ain Hospital, United Arab Emirates. We analyzed compliance against the standards set out in The National Institute for Health and Clinical Excellence (NICE) Guideline 87 concerning the diagnosis and management of ADHD in children and young people.

**Methods:**

This hospital-wide audit involved a retrospective review of case notes. A questionnaire was developed to capture the required information anonymously. The audit sample comprised 366 service users with a diagnosis of ADHD followed up in the child psychiatry clinic between January 2018 and December 2019. We chose this pre-Coronavirus Disease 2019 (COVID-19) period when services ran as usual. Data collection took place between September and November 2023.

**Results:**

Out of 366, 298 (85%) patients were males, with 181 (55%) being Emirati citizens. The sample age ranged between 5 and 17 years, with a mean of 9.8 years. Psychostimulants were prescribed in 327 (89%), with methylphenidate being the most commonly used medication. All but one case had documentation of discussion about medications' indications and side effects. However, only 40 (12%) cases had a discussion documented about the importance of following a healthy lifestyle, while 5% had no discussion about the right to revisit their decision about the treatment.

**Conclusion:**

This audit has identified areas for improvement in practice, including the need to develop local guidance on prescribing psychostimulants in children and adolescents with a diagnosis of ADHD. We recommend enhanced staff training to improve the quality of discussion with the young person and their family before starting psychostimulant therapy, including clear documentation of the decision-making process. We suggest electronic reminders to inform the patients about the importance of following a healthy lifestyle. We will re-audit the practice after one year of implementing the above action plan.

No financial sponsorship has been received for this evaluative exercise.

